# Characterization of thermochemical properties of Al nanoparticle and NiO nanowire composites

**DOI:** 10.1186/1556-276X-8-184

**Published:** 2013-04-20

**Authors:** John Z Wen, Sophie Ringuette, Golnaz Bohlouli-Zanjani, Anming Hu, Ngoc Ha Nguyen, John Persic, Catalin F Petre, Y Norman Zhou

**Affiliations:** 1 Department of Mechanical and Mechatronics Engineering, University of Waterloo, 200 University Ave. West, Waterloo, ON N2L 3G1, Canada; 2 Defence Research and Development Canada - Valcartier, 2459 Pie-XI Blvd North, Quebec, QC G3K1Y1, Canada; 3 Department of Chemistry, Center for Computational Science, Hanoi National University of Education, Hanoi, Vietnam; 4 Microbonds Inc, 151 Amber St. Unit 12, Markham, ON L3R 3B3, Canada

**Keywords:** Composite, Aluminum nanoparticles, NiO nanowires, Alumina, Nickel aluminide

## Abstract

Thermochemical properties and microstructures of the composite of Al nanoparticles and NiO nanowires were characterized. The nanowires were synthesized using a hydrothermal method and were mixed with these nanoparticles by sonication. Electron microscopic images of these composites showed dispersed NiO nanowires decorated with Al nanoparticles. Thermal analysis suggests the influence of NiO mass ratio was insignificant with regard to the onset temperature of the observed thermite reaction, although energy release values changed dramatically with varying NiO ratios. Reaction products from the fuel-rich composites were found to include elemental Al and Ni, Al_2_O_3_, and AlNi. The production of the AlNi phase, confirmed by an *ab initio* molecular dynamics simulation, was associated with the formation of some metallic liquid spheres from the thermite reaction.

## Background

Metastable intermolecular composites (MICs) are often composed of aluminum nanoparticles (the fuel is usually manufactured with a shell of alumina on each particle) and some oxidizer nanoparticles including CuO [1-12], Fe_2_O_3_[13-15], Bi_2_O_3_[5,16], MoO_3_[5,17,18], and WO_3_[5,19,20]. These MICs have drawn much attention recently in developing reliable and high-performance power generation systems due to their nanosized components which allow for the tuning of ignition temperature, reaction propagation rate, and volumetric energy density [12,17,21-24]. Applications include gas generators, micro-heaters, micro-thrusters, micro-detonators, and micro-initiators [25]. MICs can be used to fabricate an insert element which is assembled into the conventional solid propellants. This approach helps adjust ignition timing and enhance combustion propagation. However, the challenge remains in identifying a suitable MIC candidate for providing an optimal energetic performance which matches with the properties of the solid propellants.

Generally speaking, better control of the initiation process requires a sufficient heat production rate from the MIC core and a relatively slow pressure increase at the interface between the MIC core and the solid propellant. Gasless thermite reactions are desired for this reason. Gas generation from the thermite reactions is mainly attributed to the formation of vapors of metals (such as Cu, Fe, and Ni), the elemental oxygen (formed from the decomposition of the oxidizer), the gas of metal oxides if the combustion temperature is high enough, and other gaseous reaction products. While the metal vapor forms at a temperature which is above the boiling temperature of the metal, the release of elemental oxygen from the decomposition of the oxidizer component of MICs can be significant as well. Recently, Sullivan and Zachariah characterized the reaction mechanism of a variety of MICs [26], and they found that, while most oxidizers such as CuO and SnO_2_ decompose before the thermite reactions occur, which possibly indicates solid-state reactions, the decomposition of Fe_2_O_3_ becomes rate-limiting for igniting its thermite reaction. More investigations are needed in order to understand the cause of these different ignition mechanisms. Among the bulk scale thermite reactions, the Al-NiO system was reported to produce less gas [27]. Theoretically, gas (vapor and oxygen) generation from the Al/NiO thermite is about 2% of the gas produced from the Al/CuO thermite and is much lower than other comparable thermite systems. It is therefore worthwhile to investigate the thermochemical properties of the corresponding MIC made of Al and NiO nanostructures. The research objectives of this work were to synthesize and characterize the microstructures of the powder-type Al nanoparticle and NiO nanowire MIC and to investigate its ignition and energy release properties.

In the literature, there are few research papers on the characterization of Al/NiO-based composites. Recently, an Al/NiO MIC was developed on a silicon substrate [28] for fabricating a two-dimensional geometry. The process started from the thermal oxidation of a Ni film to form a NiO honeycomb. An Al layer was then coated onto this honeycomb by thermal evaporation. The produced Al/NiO MIC exhibited a low ignition temperature and improved the interfacial contact area between Al and NiO. The energy release per mass data was reported, but the method for determining that data was not reported. In that same study, the fabrication method was developed with the presence of a silicon substrate and may not be suitable for other previously mentioned applications. A more detailed investigation on thermochemical behaviors and product microstructures of the powder-type Al/NiO MIC is highly desired.

The reaction properties of a powder MIC depend on the particle size, shape, morphology, and microstructure of its fuel and oxidizer components. A variety of metal oxide nanostructures have been fabricated and implemented in developing high-energy-density MICs, which take the forms of nanospheres [29], nanowires [2,30], nanofibers [31], and nanorods [3,32]. Usually, the fineness (or particle size) and bulk density of these oxidizers and the degree of their intermixing and interfacial contacting with Al nanoparticles are among the critical factors which influence the ignition mechanism [30,33]. A recent study showed that the use of CuO nanowires resulted in better mixing between the fuel and oxidizer components of MIC and subsequently facilitated a low-temperature ignition [30]. Their measurements of the pressurization rate from a composite of Al nanoparticles and porous CuO nanowires were about ten times greater than those from the Al and CuO nanoparticle MICs. Other means such as the fabrication of the core-shell nanostructures [2,34-36] and intermetallic multilayers [22,37-39] were recently developed to enhance the energetic properties of MICs. Also, the core-shell nanowire- and nanoparticle-based thermites indeed exhibited an improved mixing homogeneity and low activation energy [2,40]. In this study, NiO nanowires were synthesized, and an effective preparation method to improve intermixing between these NiO nanowires and Al nanoparticles was developed, and then influences of the equivalence ratio of MIC on the ignition process and the energy release value were investigated. The reaction products were examined by electron microscopy and X-ray diffraction in order to identify their chemical compositions and microstructures.

## Methods

Alumina-passivated Al nanoparticles with a diameter range of 50 to 120 nm were purchased from Sigma-Aldrich Corporation (St. Louis, MO, USA). These nanoparticles were handled in an argon-filled glove box before being mixed with the oxidizer. The thickness of the oxide shell was about 5 to 8 nm which agrees with the reported data on passivated Al nanoparticles [41,42]. By assuming the averaged nanoparticle diameter of 80 nm, this shell thickness indicates that the content of Al is about 50%. NiO nanowires were synthesized by a hydrothermal method; their average diameters were approximately 20 nm, and their lengths were several microns. Hydrothermal synthesis involved two major steps. First, NiOH nanostructures were formed at 120°C in a weak alkaline solution when Ni(NO_3_) reacted with a Ni source. NiO nanowires were then produced by annealing NiOH nanostructures at 500°C for 1 h at ambient atmosphere.

The two reactants were then mixed together and ground in a 50-mL beaker in air; 10 mL of isopropanol was then added to the beaker, and the suspension was mixed in an ultrasound bath for 2 h. The suspension was then stir dried on a hot-plate stirrer. The dried powder was carefully scraped from the beaker wall and ground in an alumina mortar. Subsequently, the powder was pressed into a stainless steel die to make a pellet with a diameter of 3 mm and a height of 0.7 mm. It is worthwhile to mention that a few thermogravimetric analysis (TGA) trails were made in order to fully oxidize the Al nanoparticles in air for determining the content of Al in those particles. The results were however quite uncertain due to the low penetration of O_2_ into the core of these nanoparticles. Six different compositions indicated in Table 1 were prepared. For each composition, two samples were tested. The weight ratios of NiO in these composites were used to calculate the fuel-to-oxidizer equivalence ratio *Φ*, defined in this study by the following:

(1) $$\Phi =\frac{{\left(\frac{F}{O}\right)}_{\mathrm{act}}}{{\left(\frac{F}{O}\right)}_{\mathrm{stoi}}},$$

where ${\left(\frac{F}{O}\right)}_{\mathrm{act}}$ is the measured mass ratio of the fuel to oxidizer and ${\left(\frac{F}{O}\right)}_{\mathrm{stoi}}$ is the stoichiometric ratio calculated from the following thermite reaction between Al and NiO:

(2) $$2\mathrm{Al}+3\mathrm{NiO}={\mathrm{Al}}_{2}O_{3}+3\mathrm{Ni}\Delta H_{r}=3.4\;\mathrm{kJ}/g.$$

**Table 1:** Compositions of six Al nanoparticle and NiO nanowire composites

**Sample**	**Composition**	**Weight percentage of NiO nanowires (%)**	**Equivalence ratio ( *Φ *)**^**a**^
A	Al-NiO	9	18
B	Al-NiO	20	7
C	Al-NiO	26	5
D	Al-NiO	33	3.5
E	Al-NiO	38	2.8
F	Al-NiO	50	1.7

^a^Calculated by the Al content of 42%.

In this study, the equivalence ratios were calculated from the mass ratio of Al nanoparticles to oxidizer nanowires by taking into account the mass of the alumina shell. For this purpose, a base hydrolysis method was used to determine the amount of active aluminum in Al nanoparticles [43]. Using this volumetric technique, an amount of powder was first placed in a flask filled with a solution of 2 M NaOH. Because the active aluminum reacts with the base to form NaAlO_2_ and produce hydrogen gas, the quantity of hydrogen was measured and then used to calculate the aluminum content from the following reaction:

(3) $$2\mathrm{Al}+2\mathrm{NaOH}=2{\mathrm{NaAlO}}_{2}+3H_{2}.$$

This measurement revealed the active aluminum content of about 41% to 43%. In this study, the value of 42% was used for determining the equivalence ratio, as shown in Table 1.

The onset temperatures and energy release values were investigated by differential scanning calorimetry (DSC) and using TGA data. These tests were performed in a SDT-Q600 from TA Instruments (New Castle, DE, USA) and compared with the data from a 409 PG/PC NETZSCH (NETZSCH-Gerätebau GmbH, Selb, Germany) simultaneous thermal analysis machine which provides measurements of weight change (TGA) and differential heat flow (DSC) on the same sample. For the SDT-Q600 measurements, the DSC heat flow data were normalized using the instantaneous sample weight at any given temperature. The SDT system was calibrated by following these four steps: (1) TGA weight calibration, (2) differential thermal analysis baseline calibration for the Δ*T* signal, (3) temperature calibration, and (4) DSC heat flow calibration. In order to remove humidity, these samples were purged in argon for 15 min before thermal scanning. All DSC/TGA experiments were conducted in argon (alpha 2) with a heating rate of 10 K/min, purge flow of 50 ml/min, and temperature range between 35°C and 1,300°C. The obtained mass and heat flow signals were analyzed by the TA analysis software through which the onset temperatures and reaction enthalpies were derived. To determine the compositions of reaction products and their microstructures, the Al/NiO pellets with *Φ* = 3.5 were heated in argon to 150°C, 450°C, and 800°C on a hot plate. These experiments were performed in a glove box, and the processed pellets were then examined by scanning electron microscopy (SEM), energy dispersive spectroscopy (EDAX), and X-ray diffraction (XRD). For SEM imaging, the samples were 10 nm gold coated. The XRD patterns were captured using a Rigaku SA-HF3 (1.54 Å CuKα) X-ray source (Rigaku Corporation, Tokyo, Japan) equipped with an 800-μm collimator, operating at an excitation of 50-kV voltage, 40-mA current, and 2-kW power.

In addition, a theoretical study was conducted utilizing the *ab initio* molecular dynamics (MD) simulation to investigate the equilibrium structures of the Al/NiO MIC at different temperatures. This *ab initio* MD approach was chosen due to the lack of potentials for the Al/NiO system in the classical force field methods, such as the embedded atom model (EAM) and modified EAM (MEAN), available in the literature. To reduce the computational cost of the *ab initio* MD simulation, periodic density functional theory calculations were performed based on local density approximation and using the Ceperley-Alder exchange-correlation functionals [44]. The charge density was given by a simple summation:

(4) $$\rho \left(r\right)=\sum_{i}\left|\psi_{i}\left(r\right)\left|{}^{2}\right)\right),$$

where the summation was performed over all occupied molecular orbitals *ψ*_*i*_(***r***). The potential was calculated as follows:

(5) $$\begin{array}{l} U=\int V_{N}\left(r\right)\rho \left(r\right)\mathrm{dr}+\frac{1}{2}\int \frac{\rho \left(r_{1}\right)\rho \left(r_{2}\right)}{\left|r_{1}-r_{2}\right|}dr_{1}dr_{2}\\ \;+V_{\mathrm{NN}}, \end{array}$$

where, on the right hand side of the equation, the first term represents the electron-nucleus attraction, the second represents the electron–electron repulsion, and the final term, *V*_*NN*_, represents the nucleus-nucleus repulsion. A large-size box consisting of 25 × 15 × 12.815 Å was used, and gamma point calculations were implemented. The double zeta plus polarization basic set was employed with a very high mesh cutoff of 300 Ry. To reduce the computational cost, the norm-conserving pseudopotentials [45] were used to replace the complicated effects of the motions of the core (i.e., non-valence) electrons of an atom and its nucleus.

## Results and discussion

**Figure 1:**
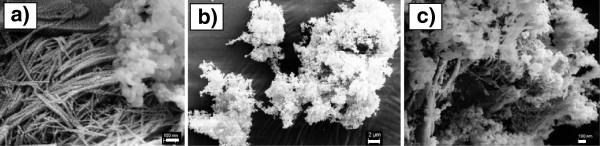
**SEM images of Al nanoparticle and NiO nanowire composites before (a) and after (b, c) sonication.** Scale bar 100 nm in (**a**), 2 μm in (**b**), and 100 nm in (**c**).

Figure 1 shows three SEM images of the mixed Al nanoparticle and NiO nanowire composite before (Figure 1a) and after (Figure 1b,c) sonication. Figure 1a demonstrates the sizes of Al nanoparticles (about 80 nm) and the diameter (about 20 nm) and length (about 1.5 μm) of NiO nanowires after mixing two components. These distinct images of two components show a poor dispersion of nanoparticles in the network of nanowires. After the solution was sonicated and dried, Al nanoparticles were able to decorate on the NiO nanowires, as shown in Figure 1b. A higher-resolution SEM image shown in Figure 1c demonstrates the nanowire branches beneath the Al nanoparticles. This process was expected to significantly increase the contact area between two components, improving thermite performance.

**Figure 2:**
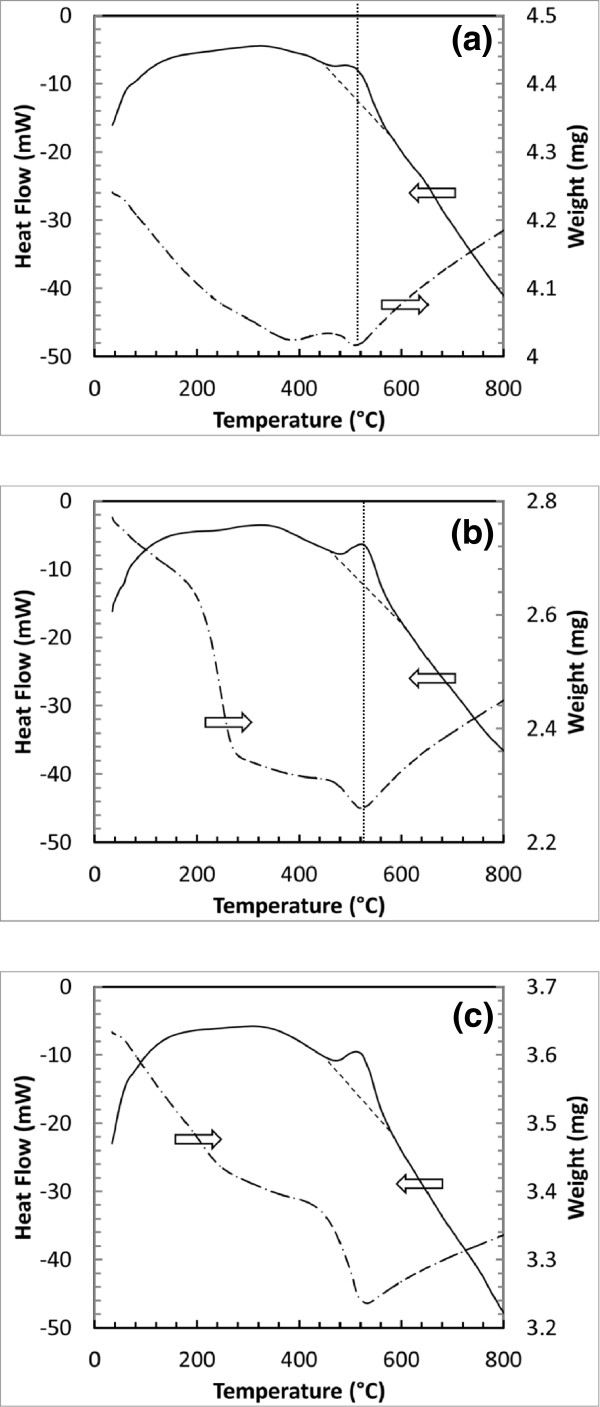
**DSC and TGA profiles measured from these Al/NiO MIC with different NiO ratios.** (**a**) Sample B 20 wt.% NiO, (**b**) sample D 33 wt.% NiO, and (**c**) sample E 38 wt.% NiO.

Figure 2 shows several DSC/TGA thermal analysis curves measured from three Al nanoparticle and NiO nanowire composites with different NiO weight ratios (for samples B, D, and E, respectively, in Table 1). Note that the heat flow curves in Figure 2a,b,c were plotted using the mass corrected values. Figure 2a was measured from sample B which originally contained about 4.2 mg of material and with a NiO weight ratio of 20%. When the sample was heated from room temperature, a slow mass loss was observed at a low temperature range (<390°C) which was attributed to the dehydration of the sample. When the temperature was increased above 400°C, the mass of the sample first increased then decreased. This behavior was associated with the mass change before and after the thermite reaction (in comparison with the heat flow curve). When the temperature is close to the onset temperature, the Al core inside the Al nanoparticles exposes to the surrounding through diffusion through or breaking the Al_2_O_3_ shell. The Al element can react with the surrounding gas such as water and oxygen if the purging flow rate is insufficient, which causes the mass increase around the ignition temperature. An obvious mass loss was observed from 450°C to 550°C, which was attributed to the formation of gas and vapor from the thermite reaction. As mentioned earlier, these gas-phase species can include vapors of metals (such as Ni) and the elemental oxygen. In this figure, the mass loss process stopped when the maximum heat flow was generated from the sample (which indicates the most energy available for vaporizing the metal products). Following the thermite reaction, the mass of the sample increased almost linearly. On the accompanying heat flow curve, the energy generation from the thermite reaction is clearly visible between 450°C and 550°C. The onset temperature was measured as 450.1°C from this curve. The area integration based on this heat flow curve provided the energy release per unit mass of the composite of about 321 J/g.

**Figure 3:**
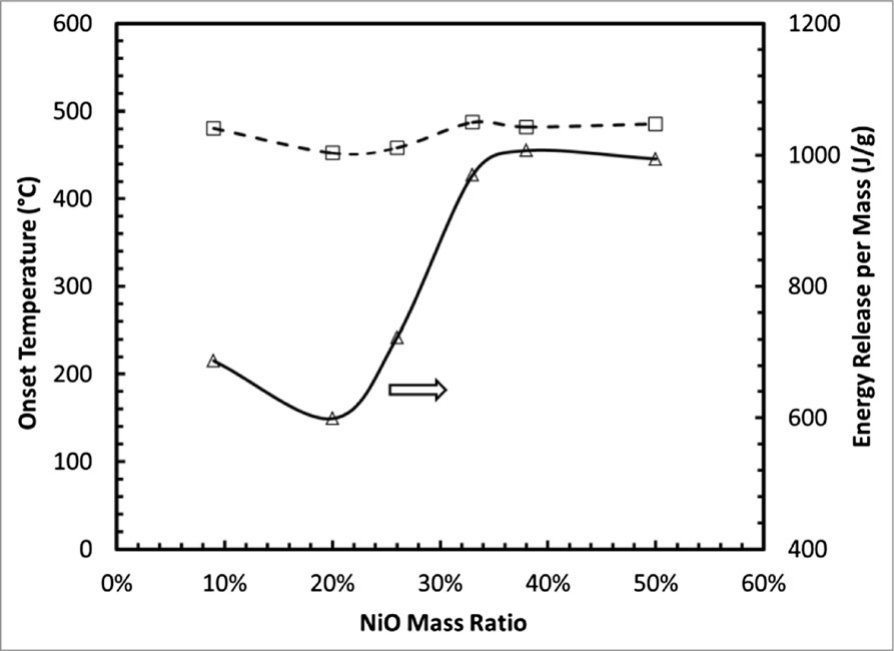
DSC-determined onset temperatures and energy release values for Al/NiO MIC with different NiO ratios.

Figure 2b shows the measured data from sample D which contained about 2.8 mg of material and with the NiO weight ratio of 33%. A multistage mass loss process was observed in the low-temperature range between room temperature and 475°C, due to hydration, and the possible decomposition of NiO. Note that for this measurement, there was little mass gain observed before the ignition of the thermite reaction, which indicates a sufficient purge process, as discussed before. A sharp mass loss was observed when the thermite reaction occurred. Again, this mass loss process stopped when the maximum heat flow was generated from the sample. On its heat flow curve, the thermite reaction was observed between 480°C and 550°C. The onset temperature for this exothermic peak was measured as 484.0°C. The energy release per mass value was determined as 593 J/g for sample D. Note that sample D produce more energy per mass due to the increased NiO amount in the composite. Figure 2c was measured from sample E which contained about 3.6 mg of material and with NiO weight ratio of 38%. The mass change and heat flow curves are very similar to these data taken for sample D. The onset temperature was measured as 475.0°C. The energy release per mass was calculated as 645 J/g. Note that the energy release values were measured by accounting for the total mass of the Al nanoparticles and NiO nanowires. Since the Al content was assumed as 42% in these Al nanoparticles, the following equation was used to determine the energy release per unit mass of the pure Al and NiO composite:

(6) $$E=\frac{E\prime \times m_{{\mathrm{Al},\mathrm{Al}}_{2}O_{3},\mathrm{NiO}}}{m_{\mathrm{Al},\mathrm{NiO}}},$$

where *E* (J/g) is the energy release per mass of MIC, *E*′ (J/g) is the DSC curve-determined energy release per mass, *m*_Al,Al2O3,NiO_ (mg) is the total mass of the composite, and *m*_Al,NiO_ (mg) is the mass of the total Al content in Al nanoparticles and NiO nanowires. Because the DSC measurements were conducted in a non-adiabatic condition, the values of *E* are much smaller than the theoretic reaction enthalpy of the reaction R2. Figure 3 shows the calculated *E* values and the measured onset temperatures from the samples shown in Table 1.

The dependence of the onset temperatures on the NiO ratios of the composites is shown in Figure 3. It can be observed that increasing the NiO ratio did not significantly change the onset temperature of the exothermic peak. This indicates a narrow size distribution of Al nanoparticles in these composites and sufficient intermixing between Al nanoparticles and NiO nanowires. All measured onset temperatures are smaller than the melting temperature of bulk Al. In the literature, it was suggested that the activation energy of the thermite reaction depends on the diffusion distance over which these metal ions (aluminum and nickel which become available from the decomposition of NiO) need to travel before initiating the reaction [46].

**Figure 4:**
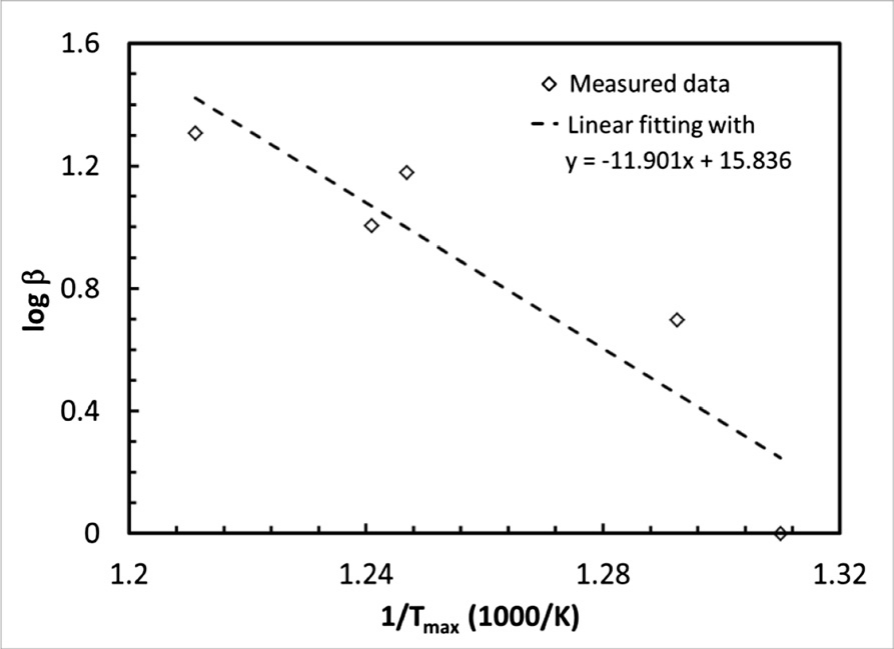
Graph used for determining the activation energy of sample D, 33 wt.% NiO, using ASTM E698 method.

To quantify the activation energy of the Al nanoparticle and NiO nanowire composites, the DSC curves of sample D was processed directly using the TA software and through the implementation of the American Society for Testing and Materials E698 method. Note that the ASTM method is often the only effective approach to analyze reactions with multiple exotherms because these peak temperatures at different heating rates are not significantly influenced by the baseline shift [47]. The ASTM E698 method generally gives an accurate assessment of the activation energy. However, calculations of the pre-exponential factor (*Z*) assume the *n*th order reaction behavior. The derived activation energies for sample D are 216.3 and 214.5 kJ/mol, respectively, from two methods. Figure 4 shows the procedure to determine the activation energy from the DSC data when the kinetic rate was expressed as a function *β*(*T*) of the temperatures *T*_max_ corresponding to the maximum heat flow. The derived activation energy agrees generally with the previously reported activation energies for Al nanoparticle-based thermite composites (such as, 248, 222, and 205 kJ/mol for the Al-Fe_2_O_3_, Al-Bi_2_O_3_, and Al-MnO_3_, respectively [48]). The activation energy of the Al nanoparticle and NiO nanowire MIC is close to but lower than the reported activation energy of the NiO reduction process (277 KJ/mol [49]). Taking into account the size effect on the reactivity of NiO nanowires, this ignition energy may indicate a thermal decomposition of NiO about the onset temperature of the studied MIC, which behaves similarly to the ignition of the Al-Bi_2_O_3_ MIC [50]. Meanwhile, for heterogeneous condensed phase MICs, the limiting factor affecting the ignition event can also be the solid-phase diffusion. Further investigations on the ignition mechanism of the Al/NiO MIC are expected.

**Figure 5:**
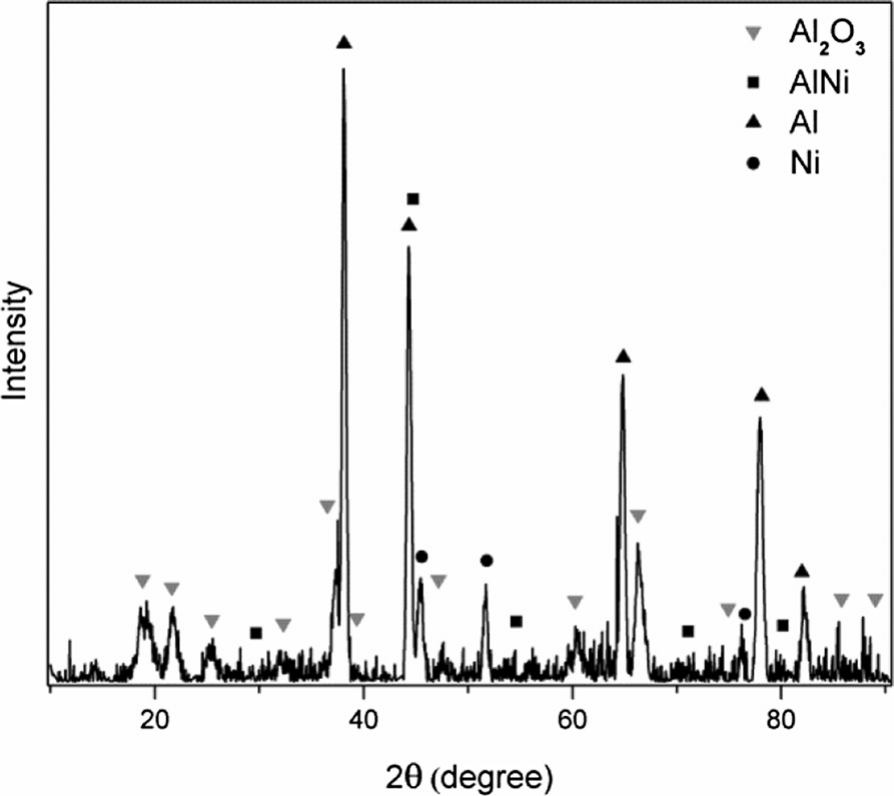
XRD patterns measured from the reaction product of sample D, 33 wt.% NiO.

The XRD analysis was performed on the reaction products from sample D which was a fuel-rich MIC with *Φ* = 3.5. As shown in Figure 5, the chemical compositions were identified as Ni, Al, AlNi, and Al_2_O_3_. Note that the identification of Al_2_O_3_ using XRD is evidential from the previous study [51]. In addition to those solid products, gaseous species such as O_2_ was also possibly formed. It is interesting to reveal the production of AlNi from the Al/NiO MIC. As a comparison, the formation of Ni was shown with lower and fewer XRD peaks, while Al still existed as a relatively large amount. Based on these observations, the following reaction was responsible:

(7) $$5\mathrm{Al}+3\mathrm{NiO}\to 3\mathrm{AlNi}+{\mathrm{Al}}_{2}O_{3}\Delta H_{r}=3.2\;\mathrm{kJ}/g.$$

**Figure 6:**
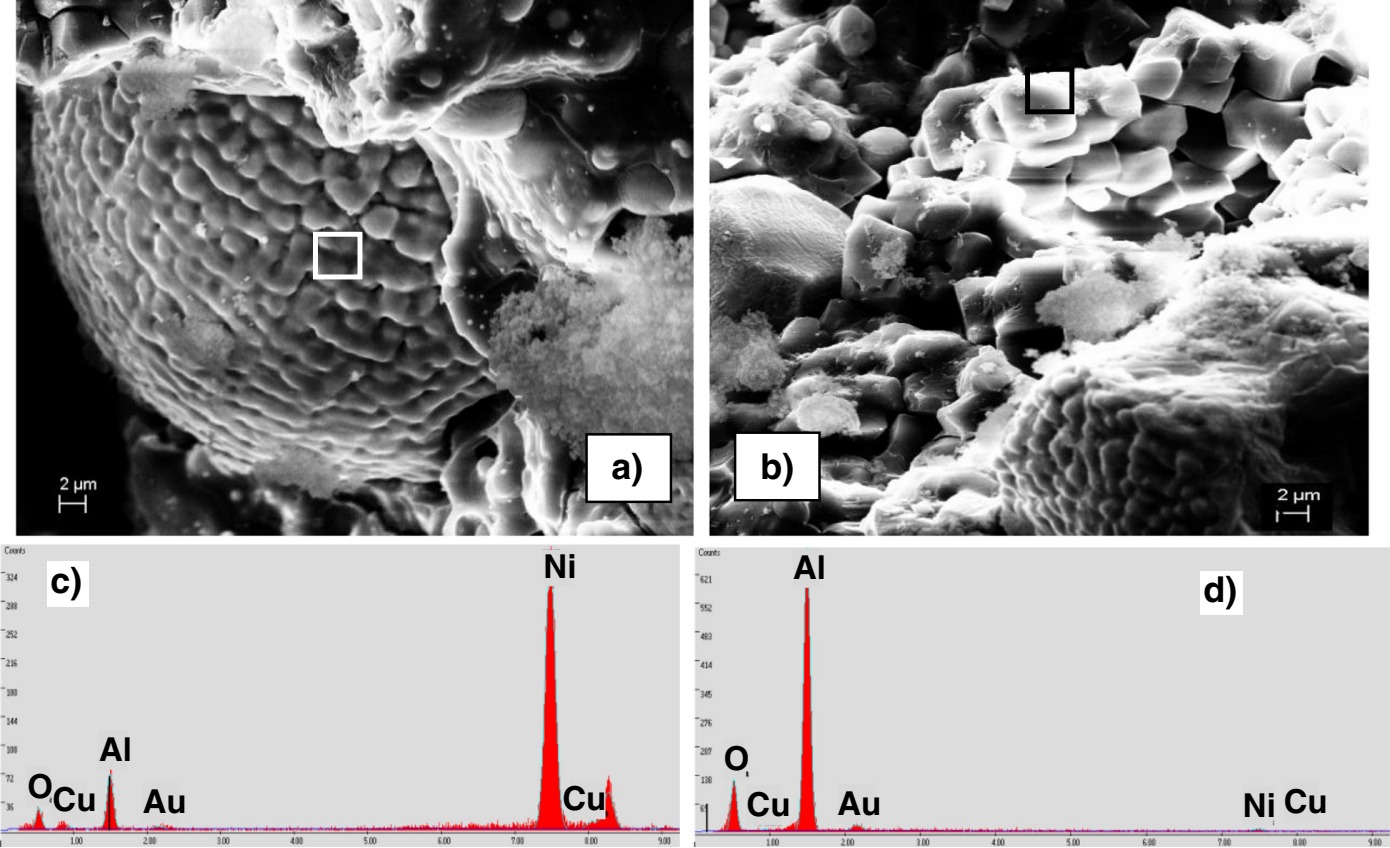
**SEM images (a, b) and respective EDX patterns (c, d).** They were obtained from the reaction products of sample D, 33 wt.% NiO.

Note that in this study, MIC *Φ* = 3.5 contained abundant Al nanoparticles and thus made the reaction R7 feasible. The propagation of R7 does not necessarily require the completeness of R2 since the decomposition of NiO may occur first and be followed by the reaction between Al and Ni. A further study on elementary reactions related to R2 and R7 is needed in order to gain more insights on this issue. To further characterize these microstructures of the products, the SEM and EDAX analyses were performed on the same product examined by XRD. Figure 6 shows two typical structures observed from MIC *Φ* = 3.5: (Figure 6a,c) a sphere which was rich in Ni and Al, and (Figure 6b,d) a bunch of Al_2_O_3_ crystalline structures. The coexistence of Ni and Al in the sphere is possibly in the form of AlNi.

**Figure 7:**
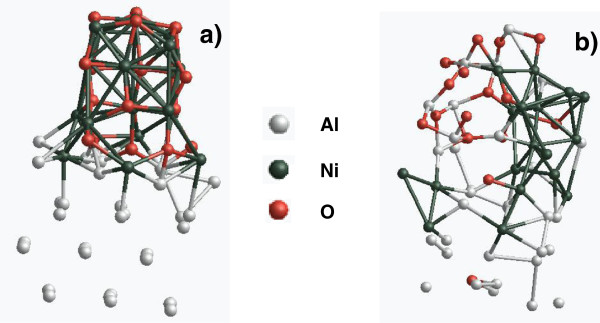
**Two MD-simulated equilibrium structures of the adjacent Al nanoparticle and a NiO nanowire.** (**a**) Temperature = 0 K; (**b**) temperature = 3,500 K.

In order to further examine the possible formation pathway of the AlNi phase, *ab initio* MD simulation was conducted for scoping the reaction time scale and identifying the equilibrium product of the thermite reaction of the Al/NiO MIC. For this simulation, the initial temperature was set to 0 K. At this temperature, the thermodynamic equilibrium structure of an Al crystalline nanoparticle and a NiO nanowire was obtained, as shown in Figure 7a. The system temperature was then increased to 1,000 K (or 726°C) to ignite the reaction. After ignition, the simulation was done under adiabatic condition. It was found that after 5 ps, as shown in Figure 7b, Al atoms diffused through the Al-NiO interface and met with O atoms (while the diffusion of O atoms into the Al nanoparticle was possible but with a much smaller chance, as observed from the image where only one O atom was found in the Al nanoparticle). Meanwhile, Ni atoms were grouped together and were intended to form the pure Ni phase. It was also observed that the AlNi phase exists at the interface between the Al nanoparticle and the NiO nanowire. Accompanying this fast thermite process, the system temperature was increased up to 3,500 K within 5 ps. This MD simulation confirmed the possibility of forming the AlNi phase from the Al-NiO thermite reaction and revealed the diffusion paths of Al and Ni atoms during the thermite reaction.

## Conclusions

In summary, the Al/NiO MIC was prepared using the NiO nanowires synthesized hydrothermally with an average diameter of about 20 nm and a length of a few microns. Six fuel-rich samples with different equivalence ratios from 1.7 to 18 were studied. The sonication process of 20 min helped produce the well-dispersed Al nanoparticles decorated on the NiO nanowires. The DSC/TGA measurements showed the onset temperatures of these Al/NiO MICs of about 460°C to 480°C. The ratio of the NiO nanowires in the MIC was found to have a less effect on the onset temperature. The derived energy release value increased significantly from 600 to 1,000 J/g when the NiO amount was increased from 9% to 50%, which were all smaller than the theoretical reaction heat of the Al and NiO thermite reaction. The chemical compositions and microstructures of these MICs were examined using XRD, SEM, and EDAX, which showed the evidence of the AlNi phase, together with the Al, Ni, and Al_2_O_3_, from the fuel-rich Al/NiO MICs. The formation mechanism of the AlNi phase was investigated using a preliminary molecular dynamics simulation which showed a diffusion of Al atoms to the Ni cluster.

## Competing interests

The authors declare that they have no competing interests.

## Authors’ contributions

JZW supervised both experimental and numerical studies and drafted the manuscript. SR, GBZ, and CFP conducted thermal analysis and other material and reaction characterization. AH performed the synthesis of nanowires. JP and YNZ co-supervised material synthesis and characterization tasks. NHN carried out the MD simulation. All authors read and approved the final manuscript.
